# Orca Behavior and Subsequent Aggression Associated with Oceanarium Confinement

**DOI:** 10.3390/ani6080049

**Published:** 2016-08-18

**Authors:** Robert Anderson, Robyn Waayers, Andrew Knight

**Affiliations:** 1 Retired, Space Dynamics Laboratory, Utah State University Research Foundation, Logan, UT 84341, USA; kin2ceta@gmail.com; 2 Palomar College, 1140 West Mission Road, San Marcos, CA 92069, USA; rwaayers@gmail.com; 3 Centre for Animal Welfare, Faculty of Humanities and Social Sciences, University of Winchester, Sparkford Road, Winchester SO22 4NR, UK

**Keywords:** orca, *Orcinus orca*, cognition, Theory of Mind (ToM), emotion, aggression, animal ethics

## Abstract

**Simple Summary:**

Orca behaviors interacting with humans within apparent friendship bonds are reviewed, and some impediments to the human evaluation of delphinid intelligence are discussed. The subsequent involvement of these orcas and their offspring in aggressive incidents with humans is also documented and examined. This is particularly relevant given that the highest recorded rates of aggressive incidents have occurred among orcas who had previously established unstructured human friendship bonds prior to their inclusion within oceanaria performances. It is concluded that the confinement of orcas within aquaria, and their use in entertainment programs, is morally indefensible, given their high intelligence, complex behaviors, and the apparent adverse effects on orcas of such confinement and use.

**Abstract:**

Based on neuroanatomical indices such as brain size and encephalization quotient, orcas are among the most intelligent animals on Earth. They display a range of complex behaviors indicative of social intelligence, but these are difficult to study in the open ocean where protective laws may apply, or in captivity, where access is constrained for commercial and safety reasons. From 1979 to 1980, however, we were able to interact with juvenile orcas in an unstructured way at San Diego’s SeaWorld facility. We observed in the animals what appeared to be pranks, tests of trust, limited use of tactical deception, emotional self-control, and empathetic behaviors. Our observations were consistent with those of a former Seaworld trainer, and provide important insights into orca cognition, communication, and social intelligence. However, after being trained as performers within Seaworld’s commercial entertainment program, a number of orcas began to exhibit aggressive behaviors. The orcas who previously established apparent friendships with humans were most affected, although significant aggression also occurred in some of their descendants, and among the orcas they lived with. Such oceanaria confinement and commercial use can no longer be considered ethically defensible, given the current understanding of orcas’ advanced cognitive, social, and communicative capacities, and of their behavioral needs.

## 1. Introduction

Intelligence is the mental quality that consists of the abilities to learn from experience, to reason, to plan and solve problems, to understand and handle abstract concepts, and to broadly comprehend and adapt to one’s surroundings [1]. Cognition includes the various mental actions and processes that underlie intelligence. While intelligence is an amorphous term with different definitions and uses by diverse groups [2], legal and social perceptions regarding animal intelligence impact animal conservation and welfare [3,4]. This paper examines orca behaviors that may be indicative of their cognitive abilities. These behaviors are presented as potential data points for the discipline of comparative cognition (Comparative cognition examines basic elements of cognition seeking to understand the nature and evolution of cognition in human and non-human animals) [5], but also as support in advocating for legislative, conservation, and welfare treatment of orcas that appropriately recognizes their cognitive capacities.

Imaging studies have shown that there is a neural distinction between the processing of physical aspects and social aspects of the world, i.e., so called physical and social cognition [6]. Physical cognition is inherently tied to the environment. The marine environment provides starkly different physical circumstances from the terrestrial.

Physical adaptation to aquatic life leads to streamlined forms that generally lack the ability to manipulate and carry tools. Added buoyancy reduces striking force while currents and the viscosity of water render the control of objects more difficult. Further, the oceanic environment is three-dimensional with potential tool materials confined to the bottom surface. By its nature, the marine environment lends less advantage for tool use as well as less opportunity for tool creation [7]. While some instances of dolphins using sponges as tools have been reported, delphinids have not developed tool-making abilities comparable to humans or apes.

On the other hand, social cognition is far more invariant regarding these physical regimes. The neural structures governing instinctive sociosexual behavior have remained all but unchanged in vertebrates for 500 million years [6], rendering social cognition a more appropriate vehicle for comparing marine and terrestrial species.

## 2. Orca and Human Phylogeny, and an Introduction to the Observed Orcas

Orcas, *Orcinus orca*, occupy all the oceans on Earth, from the Arctic to the Antarctic. Orcas belong to the order Cetartiodactyla. The early ancestors of humans and orcas were already evolutionarily separate in the Cretaceous period, some 90 million years ago. Rapid encephalization within the orca line of evolution developed during the Oligocene period; that within the human line began during the Pliocene period. Orca and dolphin lineages differentiated around 8 million years ago; human and chimpanzee lineages differentiated around 6 million years ago [8]. 

Genetic studies of orca ecotypes (An ecotype is a distinct population, adapted to a particular environment, and exhibiting behavioral and physiological differences from other members of a species) indicate that the root divergence dates back some 700,000 years [9]. *Homo sapiens* have existed for approximately 200,000 years [10]. Quite arguably, both species’ successes are based on complex social life, evolvable cultures, and intelligence [11,12].

Orcas have only been maintained in captivity starting in the 1960s when the first captives were studied and displayed in oceanaria. Orca ecotypes with oceanic coastal ranges have a long tradition of powerful totemic association to some native peoples. In Western civilization, orcas have long been considered ferocious killers as exemplified by the writings of Pliny the Elder, et al. (Naturalis Historia, IX, Chapter (5) The Natural History of Fishes, 6. The Balæna and the Orca, “This fact however is known to the orca, an animal which is particularly hostile to the balæna, and the form of which cannot in any way adequately be described, but as an enormous mass of flesh armed with teeth”. The Natural History of Pliny, Volume II, Bostok, J. and Riley H. T. (1855). Captain Robert F. Scott, RN, in his 5 January 1911 journal entry on Herbert Ponting’s encounter with orcas at the edge of an ice floe, describes the orcas as *hideous* and that their teeth are the *largest and most terrifying in the world* [13]. As recently as 1973, the U.S. Navy *Diving Manual* gave orcas their highest danger rating, referring to them as extremely ferocious beasts attacking humans at every opportunity [14] (p. 11)). 

During 1979 and 1980, the San Diego SeaWorld facility (San Diego, CA, USA) possessed a number of juvenile orcas, aged two to five years that were in training to become performers. They were in their first one to three years of captivity. These orcas were rotated one or two at a time into a marine mammal petting pool (subsequently referred to as the pool) where visitors could interact with them. Authors Anderson and Waayers were among some two dozen regular visitors who established bonds with these orcas. Waayers spent more time with the orcas than Anderson, and the authors include the experiences of others who interacted with the orcas (Alan Deeley, Russell Hockins, and Lisa Larrabee), as well, in the following. Narratives and photographs of the interactions can be found on the authors’ website: www.KinToCetaceans.org.

The orcas were named: Canuck 2 (subsequently referred to as Canuck), Katina, Kasatka, and Kotar. They were of the North Atlantic ecotype, having been captured in Icelandic waters.

This circumstance was rather unique. Orcas who had experienced one to three years of socialization in wild pods were able to form friendship bonds with humans of their own choosing and largely free from supervision. Current United States and Canadian laws forbid human interactions with and constrain approaching wild orcas. Increasing concern over aggressive and sometimes deadly human-orca incidents have led to occupational safety regulations limiting human interactions with captive orcas. Captive orcas are legally held to be property, and they are monetarily valuable. Worldwide there are over 50 orcas in captivity, of which SeaWorld Parks and Entertainment owns around 30. There are corporate protocols for their handling, training, and use in performances, described in former SeaWorld trainer John Hargrove’s book, *Beneath the Surface* [15] (pp. 43–67).

## 3. Anthropomorphism as a Tool for Cognitive Research

There is a cluster of behaviors, observable in social species that if applied to humans would designate friendship or some other affectionate bond: maintaining physical proximity, mutual grooming, affectionate contact and vocalizations, and mutual focused attention. The human emotions associated with such bonds include affection and trust. Silk discusses the use of the word friendship to describe such relations among non-human primates [16]. There are likewise behaviors associated with physical separation and reactions to a third party that if applied to humans would designate separation anxiety and jealousy.

Humans mutually engaged in these bonding behaviors develop synchronized emotional states. This is often reflected in both spoken and body languages. Humans can also develop such bonds with non-human animals; e.g., with domesticated companion animals. They mutually engage in bonding behaviors. The human experiences emotions equivalent to those in human-human bonding and can observe the body language and vocalizations of the non-human. There are numerous studies on the psychological and physiological effects on humans due to interactions with companion animals [17], as well as studies on the physiological and wellbeing effects on dogs and laboratory animals from interactions with their caretakers [18,19].

Charles Darwin believed that higher human facilities had developed gradually and that humans and other animals differed more in degree than absolute nature [20] (pp. 34–69). He made anthropomorphic assessments of animal behaviors in his writings. Subsequent scientific thought rendered the term anthropomorphism a pejorative, holding that subjective experience was outside the realm of scientific inquiry. However, scientific consensus has changed. Burghardt has advocated for a critical anthropomorphism that uses empathy and intuition to produce constantly refined and publicly verifiable predictions [21].

While the experiences that authors Anderson and Waayers had with these orcas were not controlled experiments, they might suggest hypotheses that are subject to verification. The possibility of adapting this approach for learning more about orca social cognition is discussed later in this paper. Such future examinations of orca cognition would undoubtedly be increased in scientific value by following such approaches as suggested by critical anthropomorphism.

SeaWorld orca trainers spent years working with other marine mammals before working with orcas. Then they entered an apprenticeship with a cadre of senior orca trainers prior to any physical interactions with orcas [15] (pp. 34–38). Apprentices could not even interact with an orca through a glass window, lest that interaction lead to unwanted behaviors [15] (p. 50).

In contrast, the visitors who established friendships with the orcas had no training and no surrounding cadre of experienced mentors. For their part, Anderson and Waayers followed their own empathy and intuition—their own anthropomorphic assessments of orcas. These were sufficient to establish the mutual behaviors that by subjective assessment represented friendship.

## 4. The Human-Animal Bond

Orcas are social mammals that will readily bond with humans. Humans and non-humans demonstrate corresponding behavior, body language, and vocalizations which by argument to analogy could indicate synchronized emotional states. The human ability to interpret non-human body language and vocalizations varies with learning experience. It is arguably adaptive for any animal facing either predator or prey species to have such an ability. This ability appears to be flexible in that after some exposure, the emotion being expressed (its body language representation) by the non-human may implicitly evoke the corresponding emotion in the human (depending on circumstances), without the time delay of reflection. Humans’ non-conscious perception of emotional stimuli such as facial expressions is well established, and its neuroanatomy is of ancient lineage [22]. Non-humans may implicitly perceive human body language as well, as documented by Smith et al. [23], and in the authors’ experiences as described below.

## 5. Observations of Orca Behavior and the Petting Pool Environment

Human visitors at the petting pool were restricted to leaning against an outer pool wall of waist height. The orcas could rest their heads on or completely slide out onto an underwater shelf extending inward from this wall. Human visitors could then contact the orcas’ heads, including the insides of their mouths, and their entire bodies if the orcas slid onto the shelf fully. These untrained orcas repeatedly sought human physical contact including face and head rubs, tongue rubs, and whole-body rubdowns. Typically, there was little supervision of the interactions between human visitors and the occupants of the petting pool. The following subsections describe additional interactions that are suggestive of social intelligence in orcas. 

### 5.1. Orca Self-Control and Reaction to Human Fear

The authors have no personal recollection of incidents in which a petting pool orca harmed a human visitor. They also did not observe mistreatment of the orcas by visitors. Once regular interaction began with an orca, the authors’ subjective interpretation was that the orca appeared to exercise caution such that human harm was avoided. 

It appeared that the orcas learned which of their behaviors resulted in negative human responses and avoided those behaviors in future interactions. If their motive was to experience positive interactions with humans (for relief from boredom, reduction of a sense of social isolation, etc.), they indeed achieved this.

Examples of possible orca modification of behavior to mitigate human fear include: (1) During the initial meeting between Anderson and Canuck, Anderson was wary of the orca’s massive tooth-filled mouth, and consciously kept his hands well clear of it. Canuck notably did not close his mouth until well clear of Anderson’s hands; (2) Kotar repeated this behavior on Anderson’s next visit. It was noted that after Anderson felt comfortable placing his hand inside the orcas’ mouths that they halted this behavior; (3) Kotar gently mouthed Anderson’s hand only after about 15 min of tongue rubbing and manipulation.

Orcas did sometimes close their mouths on human hands or forearms. Anderson noted that an orca only did this if the human had freely placed his or her hand in the orca’s mouth, and there was always ample time for removal of one’s hand if desired. The authors never observed an orca grasp a hand from mid-air. Also, when an orca did pull on a human hand, it always let go before actually pulling the person into the pool (Anderson discovered that upon stiffening his body, and creating a slight resistance to the pull, the orca was induced to release its grasp). Waayers noted that Kasatka was the most prone to “snappishness”, but her style involved an open-mouthed swipe near a person’s hand, with no actual harm done, interpreted by Waayers as a “leave me alone” signal.

### 5.2. Orca Tests of Human Trust

The authors noted what appeared to be a correlation between the level of trust a human showed an orca and the subsequent depth of the orca/human rapport. Seeming tests of trust on the part of the orcas included: (1) Canuck, when first encountering Anderson, suddenly and without warning emerged from beneath a bottlenose dolphin with which Anderson had been interacting, with the orca opening his mouth wide very close to Anderson’s face. Anderson interpreted this as a greeting combined with a test of trust; (2) Kotar greeted Anderson (upon his last visit with the orcas) by sliding on top of him as he bent over the pool wall, and holding him pinned until the author gave him a human style hug in return, seemingly mixing a test of trust with prank-like behavior; (3) Hockins reported Kasatka and Kotar simultaneously pulling on each of his arms, apparently mixing a test of trust with prank-like behavior. Anderson’s assessment is that the issues of trust, humor, and affection often seemed intertwined in the orcas’ actions. This could of course relate to the difficulty of a human interpreting orca intentions. Additionally, Hargrove [15] (p. 99) reports that a lesson he learned from senior trainer Ken Peters was that it aided his relationship with Kasatka to place himself in a vulnerable position with her.

There could be alternative explanations for the seeming trust/rapport connection. It is possible that human visitors that were less fearful and more inventive in their interactions with the orcas were seen to be more stimulating and thus more desirable for the orcas to interact with.

### 5.3. Limited Use of Tactical Deception

A tactical deception is an act from the normal repertoire of one individual, deployed such that another individual will likely misinterpret what the act signifies, to the advantage of the first individual [24]. Mitchell and Thompson [25] (pp. 21–29) define four levels of tactical deception. Level 4 deception involves recognition of the other animal’s beliefs about actions. Mitchell and Thompson use chimpanzees’ behavior to mislead others about the location of food as an example of this level. They describe this level as planned and self-programmed. Humans and many non-human primate species practice such deception, arguably with intent [26]. 

Orcas did not appear to display this level of tactical deception in the authors’ experience, other than its possible application for humor, as described in the next section. It should be noted that Bigg’s or Transient orcas are silent when hunting marine mammals [27], which Mitchell and Thompson [25] (pp. 21–29) would classify as Level 2 deception, i.e., coordination of perception and action. 

### 5.4. Orca Pranks

The orcas seemed inclined to play pranks, or in other words to display behaviors that appeared to have the sole intent of generating a certain desired reaction in a human or humans. Pranks and tactical deception both contain an element of instilling false beliefs; however, pranks have a humorous intent as opposed to deception’s intent to take advantage.

Tactical deception has been noted as a potential indicator of Theory of Mind (ToM) in apes [28]. ToM is the ability to infer what others might be thinking or feeling, and to predict what they might do in a given situation [29]. Could orca pranks be a comparable indicator of ToM?

Prank-like behavior observed among the orcas included: (1) Kotar sliding on top of the waist high outer pool wall and then squirting the crowd with a mouthful of water only after curious pool visitors approached him closely, as observed by Waayers when a tour group was present; (2) Waayers observed Kotar and Kasatka alternately beaching themselves on the lower pool ledge, as if to request a rubdown. As the closest orca was approached, it slid back into the water, and the other would beach itself further away, with the orcas never allowing an approach close enough for touching; (3) Katina beaching herself on top of Hockins’ forearms, holding him pinned. He reports that she made eye contact with him and subjectively assessed her message to be “I got you”. Whether this was a true “prank” or a demonstration of power is not clear. Hockin’s personal assessment was that it related to trust.

### 5.5. Orca Greeting Rituals

The greeting ritual is a form of focused attention following an absence. Most people have likely experienced the greeting rituals of a family dog or cat. Such greetings appear to be a mutual validation facilitating social cohesion. 

Incidents apparently related to orca greetings of humans include: (1) During the first visit in which Anderson directly interacted with him, Kotar was initially on the far side of the pool, scanning the surroundings in a spy-hop position, and his gaze had briefly fallen upon Anderson, moved on, and then snapped back on Anderson in a sort of “double-take” style. He then swam directly to Anderson, bypassing several other visitors around the pool’s edge. Presumably, Kotar had been the other orca in the pool on Anderson’s previous visit and had observed his interactions with Canuck. Not long after this initial greeting, Kotar presented Anderson with a fish, as described in Section 5.7. (2) As mentioned previously, after a several month absence, Kotar greeted Anderson by sliding on top of him as he bent over the pool wall. This appeared to be an exuberant show of affection at their reunion (From a private communication with Howard Garrett, a veteran orca observer and co-founder of The Orca Network, when asked whether orcas have a behavior equivalent to human hugs, (January 2016), “I think orcas do the equivalent of hugs. They make a lot of body contact almost constantly. The pattern seen most often is to swim side by side within touching range, usually two at a time but sometimes four or five may join them swimming abreast, probably touching their pec fins, as if holding hands. I’d say the answer is a resounding yes, and I don’t think it’s a stretch to say they’re showing affection.”). 

### 5.6. Empathy

Some orca behaviors seemed suggestive of empathy. There are various types of empathy based in separate neuroanatomies and which are separately activated [30]:

- Cognitive empathy has components of:
  ○ Cognitive ToM which relates to taking the perspective of another.
  ○ Affective ToM which relates to comprehending feelings in another.
- Affective empathy involves internalizing and mirroring the feelings of another.

Three examples below may provide evidence of empathy on the part of the orcas:

(1) Hockins describes an incident in which Kotar seemed to perform a test of human frailty on him. At first, Kotar bit down hard enough on Hockins to induce pain, and Hockins signaled Kotar to stop. Kotar eased the bite pressure but did not release Hockins. Then Kotar slowly reapplied the bite pressure. When it reached the limit of comfort, Hockins repeated the stop signal. Kotar let Hockins go, but (as interpreted by Hockins) registered surprise at the difference between the two bite pressures. This could be interpreted as reflecting cognitive ToM, affective ToM, and possibly affective empathy. Hockins notes that thereafter, none of the other orcas in the pool ever bit down harder than this level that Kotar established, although none of the other orcas performed this sort of test on Hockins.

(2) The authors were first drawn to the orcas in part by a desire to bond with a “fellow being”, and quickly learned that feeding them fish from the trays sold at the pool was not an effective way to achieve this. Many visitors did feed the orcas fish, and it was the authors’ impression that the orcas were in a moderately hungry state, at least while the pool was open to the public, as they readily accepted and ate fish from most visitors. Typically, the orcas would stay with most visitors just long enough to accept fish, and then move along to the next visitor. However, the orcas seemed to spend longer periods of time interacting with people who did not feed them. The authors interpret this as an effort on the orcas’ behalf to interact with certain humans at a level of friendship (as defined in Section 3), i.e., maintaining physical proximity, displaying affectionate contact and vocalization, and mutual focused attention. This could be a reflection of affective ToM (comprehending feelings in another—in this case feelings of affection directed at the orcas by humans). 

(3) Hargrove [15] (pp. 141–142) describes an accident during a performance with Takara, the daughter of Kasatka and Kotar. Immediately after the accident, Takara proceeded to use echolocation throughout his body in a way he had not experienced before. Then, Takara very gently pushed him to the edge of the pool in a way she had never been trained to do. Hargrove was later discovered to have broken ribs and soft tissue damage. This could be interpreted as reflecting cognitive ToM, affective ToM, and possibly affective empathy. 

### 5.7. Adaptation of Natural Behavior to Orca/Human Interactions

When the orca Kotar first met Anderson and Larrabee, he presented them with fish. Resident ecotype orcas share food within their pods [31], and Keiko, a North Atlantic ecotype, was reported to return fish to his rehabilitators when he was being re-introduced to catching live fish [32] (p. 241). Kotar’s act seemed symbolic and appeared to adapt a natural behavior to a novel situation with related, albeit modified, meaning. 

### 5.8. Cognitive Complexity

The orcas could quickly and smoothly switch between multiple threads of mental activity, as described in the examples below: (1) As observed by Anderson and Hockins, sometimes Kotar would interact with them for several minutes, then swim around the pool perimeter in search of offered fish, and then return for more non-food related interaction. He would repeat this cycle many times. On his first meeting with Anderson, Kotar saved the last fish from a circuit of the pool and presented it to Anderson. He repeated this behavior until Anderson understood the fish was a gift and accepted it; (2) When Hockins was being tugged on simultaneously by Kotar and Kasatka, this demonstrated coordinated actions between themselves while simultaneously interacting with a human; (3) Similarly, these two orcas alternately beaching themselves to request a rubdown but then not allowing Waayers to touch them suggests similar mental coordination.

Coordinated teamwork has been well documented and might be expected from a social predatory species such as the orca [33], and it appears that orcas can hold multiple behavioral contexts in mind simultaneously and readily switch their focus between them. Likewise, they can execute behaviors involving at least three individuals, including multiple species.

## 6. Pod Level Communications

The petting pool orcas were all captured in near coastal waters off southern Iceland in two successive years. It appears that Katina, Kasatka, and Kotar may have been captured on the same day by the same boat and crew and possibly were from the same pod. Some of the records on this are unclear. When the petting pool orcas became performers, they shared a tank with an orca named Kenau, who was captured one year prior, also off Iceland. Winston, another performing whale, was a Resident ecotype captured off the U.S. state of Washington, and so was the only non-Icelandic orca in contact with the petting pool orcas when they became performers.

The Icelandic orcas could have reasonably had prior cultural commonality as well as a not too distant familial relationship, particularly Katina, Kasatka, and Kotar. To whatever extent orcas are able to communicate, there is a good chance they had at least related dialects.

There was a degree of coherence in the behavior of these orcas that would seem to indicate they were sharing information, some of which was fairly complex. Some instances of directly coordinated behavior have already been noted as in Kasatka and Kotar simultaneously pulling Hockins toward the pool and alternately enticing Waayers for a rubdown.

Hockins noted that after Kotar performed a bite pressure test on him, no orca ever bit down harder than the limit Kotar had established as acceptable. Anderson can confirm that the three orcas who mouthed him all applied a uniformly firm but not painful pressure. As best as he can reconstruct, Anderson’s first such experience happened in the same month as Kotar’s test on Hockins but he cannot definitively say which experience occurred first.

Hargrove reported Kasatka, who was clearly the dominant orca in the “pod” when he worked with her, according to his interpretation of the events: (1) communicating with another orca that she was not visually able to see, causing that orca to reject a fish reward; and (2) inducing another orca that had been given a salmon to pass the salmon to Kasatka through a gate [15] (p. 95). He did not report a mechanism of communication (for example, prior vocalization). 

## 7. Subsequent Orca Aggression

A substantial minority of SeaWorld orcas have responded aggressively towards their trainers and other staff members. Recorded incidents include such behaviors as:

- Pushing trainers about in the water
- Mouthing, sometimes to the extent of leaving permanent bite marks, and even breaking bones
- Lunging at staff who approached the water
- Pulling trainers into the water and holding them under

The petting pool orcas and some of their offspring exhibited such aggressive incidents at a noticeably higher rate than any other grouping of incident prone orcas. The next highest rate of such incidents occurs in the group of orcas who were tank mates of the petting pool orcas during the time of visitor friendships. These tank mates were full time performers who had previously completed initial training. There are no indications that they had spent time in the petting pool. The petting pool orcas trained alongside them and did some initial public performances with them. An examination of this history potentially provides further insight into orca social cognition.

### 7.1. Post Petting Pool History

When these petting pool orcas later completed performer training, the unstructured interactions with human friends stopped. The orcas were shipped among the various SeaWorld facilities and lived the lives of full time performers. Within a few years, these orcas started to become aggressive. Hargrove [15] (p. 58) says that Kasatka was considered the most dangerous whale at SeaWorld during water work. Interestingly, Waayers observed that Kasatka was more “snappish” and more prone to being “moody” than the other petting pool orcas that she encountered, even in the pre-training days. Kasatka never inflicted physical harm during the petting pool days, though, as far as the authors know. Ultimately, these orcas became a fundamental part of a history that has significantly impacted public views in the U.S. on orca captivity. Bekoff, Anderson, Waayers, and other orca friends discussed this in a Psychology Today article [34], and on the authors’ website.

In early 2013, a documentary titled “Blackfish” premiered at the Sundance Film Festival in Park City, Utah, USA. It was subsequently purchased by Cable Network News and after some limited audience showings, it was publicly aired in late 2013 and has been shown many times since. Blackfish primarily focuses on the story of an orca named Tilikum who killed SeaWorld trainer Dawn Brancheau in 2010. Tilikum had previously been involved in the deaths of two other humans. Blackfish reviews some of the history of orca captivity and Tilikum’s earlier life at a marine park named Sealand of the Pacific in British Columbia, Canada. All this is presented against the backdrop of other aggressive human-orca and orca-on-orca incidents at SeaWorld. SeaWorld contends that Blackfish conveys falsehoods and employs emotional manipulation techniques. These differing perspectives have polarized public opinion regarding orca captivity.

Similar to an actual historical investigation, one is forced to utilize the surviving documentation and testimonies of the events to attempt to construct a rational account. As the authors do not have original source documentation, they have had to depend in some cases on the work of others. The orca incident data used in the following is based on the work of Stephan Jacobs [35]. He has compiled a database of official reports and local media articles at the time of the incidents, as well as private communications. While many reports are specific as to the date, some are remembrances of a number of incidents over a range of years. It is likely that there were additional unrecorded incidents. Jacobs was a volunteer observer of Resident and Bigg’s/Transient orcas and is mentioned in *Death at SeaWorld* [32] (p. 356).

As of this writing in May 2016 and since their founding in 1964, SeaWorld has owned 65 orcas of whom 29 (45%) have been involved in one or more published aggressive incidents. A small number of orcas account for the greatest number of incidents. The orcas with greater numbers of incidents appear to fall into five recognizable groups by background and/or ancestry. Having divided a small number of orcas into these groups, they are so few in number that no statistical significance can be claimed, only interesting tendencies. Table 1 summarizes this incident data by group. Supplementary Materials Section S1 lists by name the orcas constituting each group and the number of incidents for each orca. 

**Figure 1:**
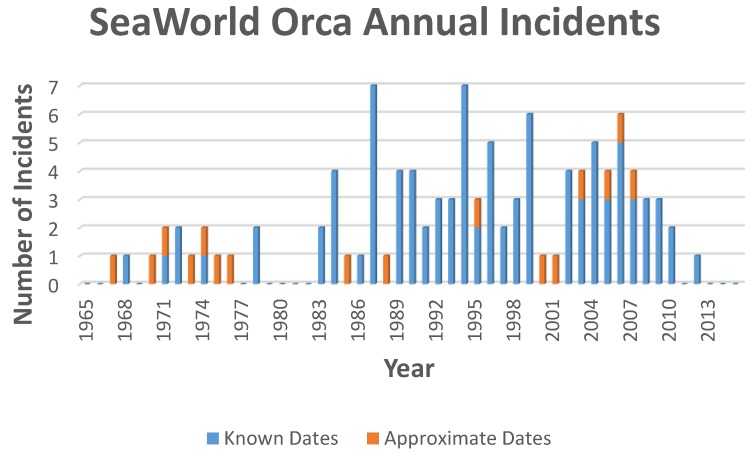
SeaWorld orca annual incidents (Some notes: The orange bars are placeholders representing non-specific entries in the database; Incidents include all those attributed to orcas that were at other oceanaria prior to arrival at SeaWorld, e.g., Tilikum’s two incidents prior to his acquisition by SeaWorld).

Summing the SeaWorld orca incident data by year reveals another interesting aspect: there are no published incidents during the petting pool years (1979 and 1980) or the following two years. Of the six incidents in 1983 and 1984, four are attributed to the petting pool orca Kandu 5 and one to tank mate Kenau. See Figure 1 below (Kandu 5 was captured in the same year as Canuck. Kandu 5 was a tank mate who had likely been a petting pool orca prior to the authors’ meeting the other four petting pool orcas).

About 55% of all of SeaWorld’s orcas have no published aggressive incidents. Of those with no incidents, nine lived beyond puberty and into at least early adulthood. An additional 15 were within the age bounds of puberty. Table 2 summarizes this information. Supplementary Materials Section S2 lists all these orcas by name, sex, and age. It also defines the age ranges used to define the maturity categories. 

By comparison, almost all of SeaWorld’s orcas with aggression lived to be sexually mature adults, i.e., 26 mature, 2 in puberty, and 1 pre-puberty. Table 3 summarizes this information. Supplementary Materials Section S3 lists all these orcas by name, sex, age, and number of incidents, with age categories for the start of puberty and sexual maturity per [36,37,38].

It should be noted that some orcas have recorded aggressive incidents starting in pre-puberty: Kayla, Keto, Orkid, Tilikum, and Taku. Kandu 5 had two recorded incidents at the very nominal beginning of puberty. Other than Tilikum, these orcas are all petting pool orcas or their offspring, or tank mate offspring, or joint Tilikum-petting pool offspring. 

Thus 26 (28) orcas with incidents lived to maturity (mature + in-puberty), while 9 (24) orcas without incidents lived to maturity (mature + in-puberty). This supports a possible correlation of orca aggression to adult versus juvenile behavior and to captive living environment. However, these correlations do not appear as strong as the correlation to having experienced unstructured interactions with visitors. The resultant aggression could still result from the combination of factors.

Of additional note, Kona 2 was captured off Iceland along with Kandu 5 and Canuck 2. She was processed with them prior to transfer to SeaWorld; however, she was instead sent to the Orlando SeaWorld facility for training. The same was true of Kahana. She was captured with Katina, Kasatka, and Kotar. She too was sent to Orlando for training.

While it is possible to discover a number of independent references to the San Diego orcas interacting with visitors, the authors have not been able to discover any reference to such a practice at Orlando. It is fairly certain that Kona 2 and Kahana did not have the opportunity to develop unstructured relationships with visiting humans. Kona 2 and Kahana both died post-puberty with no published aggressive incidents in spite of having shared the same capture and initial handling. Their cases demonstrate the contrapositive.

### 7.2. Changes To Deceptive Behavior

Hargrove [15] notes that Orkid would solicit contact with a trainer, and then strike out or grab the trainer (p. 92), or in general that orcas can quietly plot revenge, waiting the right moment to act (p. 98). These are forms of deception with apparent intent, so orcas clearly have the cognitive capacity for deception; however, as with aggression, the authors did not experience orca deception during the time of friendship. This would appear to be another facet of the change that occurred.

### 7.3. Possible Causation of the Behavioral Changes

Latham and Mason [39] examined the development of stereotypical behaviors in caged animals that were initially reared in an enriched environment, and were then removed to a standard environment. They found two divergent responses in that loss of enrichment led either to increased or to decreased development of stereotypical behaviors. They advance a “Frustration Hypothesis” to explain the former and a “Protection Hypothesis” to explain the latter. Regardless of the underlying cause they recommend caution against providing early enrichment if lifelong enrichment cannot be guaranteed.

The question then arises in the case of the petting pool orcas as to what the particular enrichment might have been. The presence of similar behavior in the offspring and tank mates of the former group of orcas potentially indicate cultural transmission of enrichments or the knowledge that they once existed.

Alternatively, increased exposure to humans at a young age may have reduced wariness of orcas around humans at later times in their lives. If this were the case, it would apply to first generation orcas exposed to enrichment, and possibly second generation orcas if this reduction in wariness was culturally transmitted.

## 8. Discussion

Much of the public and academic discussion about animal welfare has centered on the ethical concerns that arise when humans cause suffering to sentient beings. In the case of delphinids, additional questions arise regarding the level of ethical considerations humans owe to those animals closest to humans in cognitive complexity and capacity [40].

A significant impediment resides in the fact that humans and delphinids have distantly separated evolutionary histories. While the adaptive evolutionary logic of social life was common to both, the evolutionary solutions were often unique, especially in that the solutions were subject to the peculiarities of terrestrial versus marine environments.

Marino [8] discusses the evolution of intelligence by comparing primates and cetaceans. She notes their distantly separated evolutionary lines, and that being mammals, they share subcortical neuroanatomy but are radically divergent in cortical development. She notes striking convergence in a number of behavioral and social complexity realms, and also notes that it is still to be seen whether such convergence exists at more abstract levels, such as, introspection, mental state attribution, deception, moral judgment, etc.

The Encephalization Quotient (EQ) provides one measure of intelligence. The Appendix A discusses EQ and has a table showing EQ for orcas and various other mammals, including chimpanzees.

Consistent with Marino’s note on shared mammalian subcortical neuroanatomy, the authors of this paper found orcas quite emotionally familiar, as orcas seem to have done with humans. Additionally, the authors have begun addressing Marino’s question on those more abstract levels as described above with orcas. The quest to objectively compare delphinid cognitive complexity and capacity (or “intelligence”) to that in humans is however made difficult by the non-overlap of capabilities that humans consider to be benchmarks of intelligence.

### 8.1. Common Theory of Mind Scenarios and Delphinids

Often, comparisons of human and non-human ToM abilities involve false beliefs; e.g., deceptive behaviors in chimpanzees regarding the location of some food item and the potential knowledge of a competitor regarding that food [28]. Another common scenario is caching and retrieval of food items by scrub jays demonstrating knowledge of a competitor’s mental representations [41]. In both cases, the visual lines of sight of subject and competitor are often a significant consideration.

Orcas appear to practice only low-level deception, as in silent hunting by Bigg’s orcas. The authors’ only observations of deception in orcas related to their tendency towards humorous pranks.

Orcas practice food sharing among themselves. They do not cache food or other objects. They do not accumulate or carry about objects, except very temporarily in their mouths. As apex predators, they have little necessary concern about other species stealing their food.

Orcas have excellent eyesight, but much of their lives are spent in murky waters and sometimes at depths where lighting is greatly reduced. They are highly dependent on echolocation, a sensory mode that humans scarcely possess [42]. The physical properties of underwater acoustics make it feasible that orcas possess a sense of something like three-dimensional sonograms. Of course we do not know what their sensory perception might actually look like. 

Of note, the acoustic pulses of echolocation pass through solid objects that would be opaque to light. The echoes occur at material boundaries where the properties of acoustic transport change. Some acoustic energy is reflected while other energy continues to propagate through the new material.

Unlike humans, orcas have two modes of perception with quite different characteristics regarding whether an object would be hidden behind another. Any comparison of ToM and other complex cognitive abilities in humans and orcas needs to consider that behaviors or environmental factors critical to one species might be moot to the other.

### 8.2. Thoughts on Future Study of Orca Cognition

Dolphins have been studied since the 1960s but certainly less extensively than chimpanzees. Their physically impressive cousins, orcas, have been even less studied. 

As explored earlier in this paper, orcas and humans have significant overlaps in emotion and social cognition. Humans and orcas can develop friendships. While the friendships developed in 1979–1980 were unstructured, they still enabled significant insights into orca social cognition. If human-orca friendships were approached in a more integrated fashion, for instance, a cultural immersion such as Jane Goodall experienced with chimpanzees, it is possible that far more could be learned.

SeaWorld announced in March 2016 that it would cease breeding its orcas and no longer have orcas performing in theatrical shows. SeaWorld stated in a press release, “As society’s understanding of orcas continues to change, SeaWorld is changing with it”. Since the release of Blackfish and facing a continuing campaign by People for the Ethical Treatment of Animals (PETA), SeaWorld’s attendance and stock prices have been suffering.

As two of the original petting pool orcas still survive, potential friendships could be revived, and those two orcas could form the basis for extending the friendships to other orcas. The authors suggest that a resumption of treating the orcas with basic respect and rebuilding trust are necessary first steps.

Establishing communications with delphinids in some form of common language would be a fundamental breakthrough. Direct human-delphinid communications could lead to more popular acceptance of delphinids as deserving moral consideration, more than any other revelation. Researchers have been seeking this goal since the 1960s. While advances have been made, incontrovertible results have been elusive. 

The authors suggest that achieving communication breakthroughs could well depend on thinking about how information might be encoded utilizing the physically possible means available to delphinids. Humans encode speech on a single channel that is amplitude and frequency modulated. Information is presented sequentially such that a syntax is required to determine the meaning of word sequences, e.g., for English: subject, verb, and object.

Delphinids have at least three channels. They have two phonic lips that can generate different sound types either completely independently, e.g., whistles and clicks, or in phase conjunction with each other [43] (pp. 302–323). A third channel exists in that the acoustic energy is focused within a steerable beam of about a 10° width. Hargrove reported that he could feel Takara’s echolocation pulses within his body [15] (p. 141). A dolphin’s facial area is highly innervated, about equivalent to human fingertips [44]. This is believed to be an aid in locating objects close to their mouths by pressure waves in the water, as in catching fish. The authors suggest it would be worthwhile to investigate whether the steerable acoustic beam of one dolphin impinging on the facial area of another dolphin might be used to encode and transfer information.

Dolphin whistles have already been associated with identities [45]. The authors suggest the possibility that different parts of a delphinid “sentence” could be transmitted in parallel on the different physically available channels. This would obviate the need for some of the syntactic rules humans require to decode their purely sequential verbal expressions.

The principal reason humans have not succeeded in understanding delphinid communications during the preceding 50 years may relate less to the technology applied or that is available, but more to the human tendency to limit thinking to particularly human solutions. Perhaps achieving better understanding of the so familiar yet so alien orca could help free humans of this anthropocentric tendency.

## 9. Conclusions

In various parts of the world, human-orca relations have been and continue to be dominated by abusive and exploitative capture, confinement and training methods, and by the use of orcas in entertainment programs within oceanaria. The Whale and Dolphin Conservation Society [46] notes that captive orcas are confined within oceanaria in Argentina, Canada, China, France, Japan, Russia, Spain, and the United States. Russia also has an active orca capture industry. SeaWorld’s recent announcements of the cessation of captive orca breeding and the use of orcas in theatrical performances are promising. However, the orca Lolita, also called Tokitae, is still a performing captive at the Miami Seaquarium in the United States. This is in spite of Lolita being a Southern Resident orca, an officially recognized endangered ecotype.

In light of these facts, the significant cognitive capabilities of orcas clearly indicated by the experiences of ourselves and others are of profound ethical weight. We would do well to appreciate not only that orcas and dolphins are the closest non-humans to ourselves in EQ (and some mental attributes similar to humans), but also that the limits of their intelligence are not yet clearly defined. We would be similarly wise to appreciate that orcas’ rise to intelligence preceded that of modern humans by more than twice the time that our own species has existed. In fact, orcas were very likely among the most dominant predators in the world’s oceans, before *Homo sapiens* even left Africa. Given these insights, we can no longer ethically continue to treat these remarkable creatures as we have done. Instead, a fundamental refocusing of our relationships with orcas is warranted, in favor of a new era characterized by mutual friendship, understanding, and much greater appreciation of these remarkable creatures than has been the case to date.

## Figures and Tables

**Table 1:** Summary of orca incidents by group.

Average Incidents Per Orca */Average Incidents Per Orca **	Group	Number in Group with Incidents/All Orcas in Group	Percent with Incidents
7.57/3.31	Petting Pool Orcas and Offspring	7/16	44%
3.67/2.20	Tank Mates and Offspring	3/5	60%
3.50/3.50	Orcas originally at Marineland	2/2	100%
2.40/1.50	Sealand Orcas and Offspring	5/8	62%
2.00/0.67	Joint Offspring of Tilikum and Petting Pool Orcas	4/12	33%
1.62/0.59	Orcas with no Known Relationships	8/22	38%

* Only including orcas with incidents; ** For all orcas in the group with and without incidents.

**Table 2:** Orcas with no reported incidents.

Sex	Maturity	Number in Group
M	Sexually Mature	3
F	Sexually Mature	6
M	In Puberty	4
F	In Puberty	11
M	Pre-puberty	5
F	Pre-Puberty	7

**Table 3:** Orcas with reported incidents.

Sex	Maturity	Number in Group
M	Sexually Mature	13
F	Sexually Mature	13
M	In Puberty	0
F	In Puberty	2
M	Pre-puberty	0
F	Pre-Puberty	1

## Supplementary Materials

The following are available online at www.mdpi.com/2076-2615/6/8/49/s1, Section S1: Orca Relationships and Aggressive Incidents, Section S2: Age Distribution of Orcas with No Published Incidents, Section S3: Age Distribution of Orcas with Published Incidents.

## Abbreviations

The following abbreviations are used in this manuscript:

DOAJ	Directory of open access journals
EQ	Encephalization Quotient
F1	First generation descendant
F2	Second generation descendant
MDPI	Multidisciplinary Digital Publishing Institute
RN	Royal Navy
ToM	Theory of Mind
UK	United Kingdom
USA	United States of America

## Appendix A

Encephalization quotient (EQ) attempts to quantify mammalian intelligence with a single numeric comparative value. EQ is a unitless number that is the ratio of actual brain mass to an estimated brain mass. The estimated brain mass is calculated using a non-linear relationship derived from an empirical fit to the data of a sample group of mammals. Some published numbers are shown in Table A1.

**Table A1:** Encephalization quotient. Data are from Roth and Dicke [47] except for orcas. The lower orca EQ is from Marino [48], and the higher orca EQ is from Woods and Evans [49].

Encephalization Quotient	Mammal
7.4 to 7.8	Human
5.3	Bottlenose Dolphin
2.57 to 2.9	Orca
2.2 to 2.5	Chimpanzee
1.3	Elephant
0.9	Horse

By this measure, dolphins and orcas are among the closest if not the closest mammals to humans in intelligence. Wood and Evans [49] give an EQ of 5.9 for the bottlenose dolphin and note that the number would be even higher if blubber were accounted for. Blubber adds to body mass but doesn’t require much neural capacity to manage. They also state that for orcas, the computed EQ is undoubtedly too low and give rationale of this assessment. Marino [50] makes a similar statement in regards to sperm and baleen whales (Mysticiti), i.e., that the scaling rules regarding brain and body size that underlie EQ do not hold. The elephant, which can pass the mirror test and is generally held to be quite intelligent, has an EQ of 1.3.

The scaling rule changes for mammals with body masses greater than 1000 kg. The largest male orcas have mass upwards of 10,000 kg. A regression based solely on mammals in excess of 1000 kg could provide a new scaling rule, but the determining data set would be small. Perhaps that data set could be extended using brain and body mass estimates from the skeletal remains of extinct Pleistocene and Pliocene megafauna.

Authors Anderson and Waayers had experiences interacting with both orcas and bottlenose dolphins. Although there were behavioral and cultural differences, nothing stood out as indicating one or the other was more intelligent. In contrast, Anderson and Waayers each noted that the pilot whale who shared the pool with the orcas was not as aware of the need to be gentle with humans.

Another factor of orca intelligence is that orcas are born precocious. Some orcas have survived entirely by themselves at less than two years of age (A Southern Resident orca designated L-98 was born in September 1999 but disappeared and was thought dead. A solitary calf was observed in the summer of 2001 in Nootka Sound off Vancouver Island, British Columbia, Canada and by the fall of 2001 had been identified as L-98. This orca, popularly called Luna, attempted to socialize with humans, which was problematic since under Canadian law such fraternization was illegal. L-98 survived on his own until 2006 when he was accidentally killed by a tugboat [51]. Other such cases are known, e.g., A-73 “Springer”). Even though the petting pool orcas were between two and five years of age, their mental maturity appeared roughly equivalent to a human at the age of mid-to-late teens.
