# Supplementary material for: Orca Behavior and Subsequent Aggression Associated with Oceanarium Confinement
Source: Animals (Basel). 2016 Aug 18;6(8):49. doi: 10.3390/ani6080049 (PMC4997274; doi:10.3390/ani6080049)
Supplement: Supplementary file 1 [file animals-06-00049-s001.pdf]

Supplementary Materials: Orca Behavior and Subsequent Aggression Associated with Oceanarium Confinement

Robert Anderson, Robyn Waayers and Andrew Knight

Section S1. Orca Relationships and Aggressive Incidents

SeaWorld purchased four orcas from Sealand of the Pacific. Sealand stopped operating after Tilikum and his pool mates killed trainer Keltie Byrne in 1991. The history depicted in *Blackfish* from interviews with former staff and visitors is that the orcas were trained in an abusive fashion and spent most of each day confined to small metal cages that they were induced to enter using food deprivation. Tilikum's offspring born at SeaWorld and unrelated to the petting pool orcas are included in the following group shown in Table S1. Tilikum arrived at SeaWorld in 1992. Nootka 2, Kyuquot, and Haida 2 arrived at SeaWorld in 1993.

Table S1. Sealand orcas and offspring.

Incidents	Orca	Relationship
5	Tilikum	
3	Nootka 4	
2	Kyuquot	F1 born at SeaLand
1	Haida 2	
1	Tekoa	F1 born at SeaWorld
0	Nyar	F1 born at SeaWorld
0	Sumar	F1 born at SeaWorld
0	Malia	F1 born at SeaWorld

Average: 2.40 incidents per orca with incidents, 1.50 average incidents per all orcas in the category, 62.5% of orcas in the category have incidents.

The following grouping, shown in Table S2, are the orcas known to the authors and also Kandu 5. Their offspring not related to Tilikum are included.

Kandu 5 was captured the month prior to Canuck 2, and they were shipped together and trained together at San Diego. She completed training before Canuck and was a full time performer when the authors became friends with the orcas. It is believed that she had also interacted with visitors, but the authors have not yet found definitive proof. She was in either case a pool mate.

Keto killed trainer Alexis Martinez in 2009 at Loro Parque in the Canary Islands. SeaWorld owns the orcas at Loro Parque. Keto is also a grandson of tank mate Winston.

The orcas in the following group, shown in Table S3, were all born in captivity at SeaWorld. They are all sons or daughters of Tilikum and child or grandchild of Katina. Tuar and Skyla are grandchildren of tank mate Winston.

The following group, in Table S4, shows orcas who were tank mates of the petting pool orcas during the time of interactions with visitors. Kayla is the daughter of Kenau and Marineland orca Orky 2.

SeaWorld acquired Winston from Windsor Safari Park in Berkshire, UK, where he had lived for 6 years. Two of Winston's incidents occurred prior to his arrival at SeaWorld.

SeaWorld bought Marineland of the Pacific, California, USA, and acquired their orcas, as shown in Table S5. Orky 2 is the father of tank mate offspring Kayla. They became tank mates to the petting pool orcas at San Diego but only beginning in 1987. Kayla could reasonably be placed in either Tables S4 or S5. Table S4 seems to make more sense in that Kenau's early years were as tank mate to Kasatka and Kotar at San Antonio. Corky 2 and Orky 2 arrived at SeaWorld in 1987.

Table S2. Petting pool orcas and offspring.

Incidents	Orca	Relationship
17	Orkid	F1
12	Kasatka	
7	Kandu 5	
6	Keto	F1/F2
5	Katina	
3	Takara	F1
3	Kotar	
0	Canuck 2 (one of the original petting pool orcas, died while still in training at less than 6 years of age and before any of the other petting pool orcas had exhibited aggression)	
0	Katerina	F1
0	Kalina	F1
0	Makani	F1
0	Kamea	F2
0	Keet	F1/F2
0	Kalia	F3
0	Victoria	F3
0	Amaya	F4

Average: 7.57 incidents per orca with incidents, 3.31 average incidents per all orcas in the category, 43.8% of orcas in the category have incidents.

Table S3. Joint offspring of Tilikum and petting pool orcas.

Incidents	Orca	Relationship
4	Tuar	F1/F2
2	Taku	F1
1	Ikaika	F1
1	Skyla	F1/F2
0	Nakai	F1
0	Makaio	F1
0	Unna	F1
0	Nalani	F1/F3
0	Trua	F3
0	Kohana	F1/F3
0	Sakari	F1/F3
0	Adan	F3

Average: 2.00 incidents per orca with incidents, 0.67 average incidents per all orcas in the category, 33.3% of orcas in the category have incidents.

Table S4. Tank mates and offspring.

Incidents	Orca	Relationship
6	Kayla	F1
3	Winston	
2	Kenau	
0	Shawn (Shawn died during her first year of captivity at less than 2 years of age)	
0	Halyn	F2 Tank Mate, F3 petting Pool

Average: 3.67 incidents per orca with incidents, or 3.0 excluding Winston's incidents prior to SeaWorld, 2.20 average incidents per all orcas in the category, 60.0% of orcas in the category have incidents.

Table S5. Orcas originally at Marineland.

Incidents	Orca	Relationship
4	Corky 2	
3	Orky 2	

Average: 3.50 incidents per orca, or 3.0 excluding the incident prior to SeaWorld, 100.0% of orcas in the category have incidents.

The following orcas, shown in Table S6, are not known to be related and their incidents occurred across a wide range of times. The only possible exception is Splash. SeaWorld acquired captive born Splash from MarineLand in Ontario, Canada. He was sent to San Diego where he was tank mates with petting pool orcas, and others. His single incident occurred in conjunction with Orkid where they dragged a trainer into the pool and broke her arm.

Table S6. Orcas with no known relationships.

Incidents	Orca	Relationship
3	Shamu	
3	Ulyses	
2	Nootka	
1	Kanduke	
1	Kandu	
1	Taima	
1	Shouka	
1	Splash	
0	Canuck	
0	Frankie	
0	Kilroy	
0	Kona	
0	Sandy	
0	Kandu 3	
0	Ramu	
0	Kona 2	
0	Gudrun	
0	Kahana	
0	Morgan	
0	Samoa	
0	Winnie	
0	Bjossa	

Average: 1.62 incidents per orca with incidents, 0.59 average incidents per all orcas in the category, 38.1% of orcas in the category have incidents.

Section S2. Age Distribution of Orcas with No Published Incidents

Table S7 shows the current age or age at death of orcas with no published incidents.

Table S7. Orcas with no published incidents.

Orca	Sex	Current Age/Age at Death
Keet	M	23.3
Ramu	M	18.9
Nakai	M	14.7
Kilroy	M	12.5
Sumar	M	12.3
Trua	M	10.5
Frankie	M	9.5
Canuck 2	M	5.8
Makaio	M	5.6
Adan	M	5.6
Canuck	M	4.7
Makani	M	3.3
Kalina	F	25.0
Bjossa	F	23.9
Gudrun	F	20.2
Unna	F	19.4
Kohana	F	14.0
Kahana	F	13.6
Kona 2	F	12.0
Kalia	F	11.4
Samoa	F	11.3
Sandy	F	10.6
Katerina	F	10.5
Kona	F	10.2
Nalani	F	9.7
Malia	F	9.2
Morgan	F	8.9
Kandu 3	F	6.8
Sakari	F	6.4
Winnie	F	5.4
Halyn	F	2.7
Kamea	F	2.4
Nyar	F	2.3
Shawn	F	1.9
Amaya	F	1.5
Victoria	F	0.9

Section S3. Age Distribution of Orcas with Published Incidents

Table S8 shows the current age or age at death of orcas with published incidents.

Male orcas [1]:

- Puberty occurs between ages 8 to 12 years
- Sexual maturity is age 13 years or greater

Female orcas [2,3]:

- Puberty occurs between ages 6 to 13 years
- Sexual maturity is age 13 years or greater

Table S8. Orcas with published incidents.

Orca	Sex	Current Age/Age at Death	Number of Incidents
Ulises	M	38.5	3
Tilikum	M	35.5	5
Orky 2	M	26.5	3
Kyuquot	M	24.4	2
Keto	M	20.9	6
Winston	M	19.7	3
Kanduke	M	19.1	1
Kotar	M	17.3	3
Tuar	M	16.9	4
Splash	M	15.6	1
Tekoa	M	15.5	1
Taku	M	14.1	2
Ikaika	M	13.7	1
Corky 2	F	49.4	4
Katina	F	40.6	5
Kasatka	F	38.6	12
Orkid	F	27.6	17
Kayla	F	27.5	8
Takara	F	24.8	3
Nootka	F	24.0	2
Shouka	F	23.2	1
Taima	F	20.9	1
Haida 2	F	19.7	1
Kenau	F	15.8	2
Kandu 5	F	14.9	7
Nootka 4	F	14.9	3
Skyla	F	12.3	1
Shamu	F	9.8	3
Kandu	F	5.0	1

References

1. Robeck, T.R.; Monfort, S.L. Characterization of male killer whale (*Orcinus orca*) sexual maturation and reproductive seasonality. *Theriogenology* **2005**, *66*, 242–250.
2. Olesiuk, P.F.; Ellis, G.M.; Ford, J.K.B. *Life History and Population Dynamics of Northern Resident Killer Whales (Orcinus orca) in British Columbia*; Canadian Science Advisory Secretariat: Nanaimo, BC, Canada, 2005.
3. Robeck, T.R.; Willis, K.; Scarpuzzi, M.R.; O'Brien, J.K. Comparisons of life-history parameters between free-ranging and captive killer whale (*Orcinus orca*) populations for application toward species management. *J. Mammal.* **2015**, *96*, 1055–1070.

© 2016 by the authors; licensee MDPI, Basel, Switzerland. This article is an open access article distributed under the terms and conditions of the Creative Commons by Attribution (CC-BY) license (http://creativecommons.org/licenses/by/4.0/).
